# A Pilot Study on Understanding the Contextual Factors Impacting the Implementation of an Antibiotic Stewardship Program in a Single Health Center Serving Rural and Underserved Communities in the United States—A Mixed-Methods Approach

**DOI:** 10.3390/antibiotics14030263

**Published:** 2025-03-05

**Authors:** Arinze Nkemdirim Okere, Anthony Ryan Pinto, Sandra Suther

**Affiliations:** 1 College of Pharmacy, The University of Iowa, 180 South Grand Ave., 366B College of Pharmacy Building (CPB), Iowa City, IA 52242, USA; 2 Community Health Northwest Florida Community-Based Pharmacy Residency Program, Florida A&M University, 2315 W Jackson St, Pensacola, FL 32505, USA; anthony.pinto@famu.edu; 3 Institute of Public Health, College of Pharmacy and Pharmaceutical Sciences, Florida A&M University, 1415 Martin Luther King Jr. BLVD, Tallahassee, FL 32307, USA; sandra.suther@famu.edu

**Keywords:** antibiotic stewardship, antibiotic, rural health, underserved population

## Abstract

**Objective**: This study aimed to identify contextual factors influencing the implementation of an antibiotic stewardship program (ASP) in a rural primary care center serving underserved communities. **Methods**: A mixed-methods approach guided by the Consolidated Framework for Implementation Research (CFIR) was employed. Data were collected through semi-structured interviews, focus groups, and surveys with clinical staff and leadership at a Federally Qualified Health Center (FQHC). The CFIR framework was used to explore barriers and facilitators within the clinic’s inner and outer settings, focusing on staff perceptions, challenges, and readiness for ASP implementation. **Results**: Strong staff support for ASPs was identified, with participants emphasizing their potential to improve patient outcomes and antibiotic prescribing practices. Barriers included insufficient training, a high workload, and patient pressure to prescribe antibiotics. Leadership commitment, enhanced communication systems, and tailored educational materials were identified as critical facilitators for successful implementation. Participants highlighted the need for accessible educational tools and streamlined protocols to improve engagement and compliance. **Conclusions**: Implementing an ASP in rural and underserved settings is feasible but requires addressing site-specific challenges. The insights from this study underscore the importance of understanding contextual factors to inform evidence-based strategies for ASP adoption. The structured use of CFIR provided a comprehensive framework to guide implementation efforts, ultimately supporting better antibiotic use and public health outcomes in resource-constrained healthcare settings.

## 1. Introduction

Patients in rural and underserved communities face a disproportionate burden of antibiotic-resistant infections (ARIs) due to inappropriate antibiotic prescribing practices [1]. These patients are also more likely to experience severe adverse health outcomes compared to their urban counterparts [1]. With over 60% of rural U.S. residents relying on rural health clinics (RHCs) for healthcare services, addressing ARIs is a critical priority for policymakers [2].

Nationwide, more than 5200 RHCs provide care to approximately 37.7 million patients annually, representing over 11% of the total U.S. population and 62% of the 60.8 million people living in rural areas [2]. This underscores the essential role RHCs play in rural healthcare and their potential to reduce ARIs through targeted interventions such as antibiotic stewardship programs (ASPs).

Despite evidence demonstrating that ASPs effectively reduce ARIs by improving antibiotic prescribing practices [3], their implementation in RHCs remains both challenging and infrequent. Recent studies have highlighted key barriers to ASP implementation, underscoring the need for targeted strategies to address these challenges in resource-limited settings [4]. However, limited research has examined the barriers, facilitators, and effective implementation strategies for antibiotic stewardship programs from both patient and organizational perspectives in rural clinics or clinics serving underserved communities in the United States.

To address this gap, we first examined rural patients’ knowledge and attitudes regarding antibiotic resistance, stewardship programs, and pharmacist involvement in prescribing antibiotics. Our findings reveal that while over 60% of respondents could name their prescribed antibiotics, fewer than 30% understood the indications or duration of their treatment. Only 41.8% recognized antibiotic resistance as a nationwide issue, and many were unaware of the risks of inappropriate antibiotic use [5]. Notably, patient awareness of antibiotic resistance positively correlated with comfort in pharmacist involvement in prescribing (r = 0.23, *p* < 0.01). Similarly, awareness of antibiotic risks showed a moderate positive correlation with comfort in pharmacist involvement (r = 0.38, *p* < 0.01) [5]. Deductively, our findings provide insights into the factors influencing the implementation of antibiotic stewardship from patients’ perspective. Specifically, the level of patient knowledge regarding antibiotic resistance and stewardship emerged as a key determinant, with gaps in understanding serving as barriers to effective implementation [5].

Building on this understanding of patient perspectives, we sought to examine the facilitators and barriers to implementing antibiotic stewardship from the clinic’s perspective. A comprehensive understanding of these factors is essential for the successful implementation of ASPs in clinics serving rural or underserved communities. Hence, this study aimed to identify the contextual factors that rural clinical staff and clinicians perceive as influencing ASP implementation in RHCs. By understanding these factors at the organizational level, we can develop and deploy a coordinated bundle of evidence-based strategies to effectively implement ASPs in this critical healthcare setting.

## 2. Methods

### 2.1. Study Design

In this cross-sectional survey, we used a mixed-methods approach to identify factors impacting the implementation of ASP in clinics serving patients residing in underserved and rural communities. Contextual factors are defined as conditions or variables influencing the success of evidence-based interventions. This study, conducted between November 2023 and January 2024, involved semi-structured interviews guided by the Consolidated Framework for Implementation Research (CFIR) [6]—a framework that systematically assesses contextual factors influencing the implementation of evidence-based practices across diverse settings. Our focus was on both the outer and inner setting components of CFIR—these components describe factors at the external and organizational levels [6,7]. This study was approved by the Institutional Review Board.

### 2.2. Setting

This study was conducted at a Federally Qualified Health Center (FQHC), a federally funded nonprofit clinic serving medically underserved and rural populations. FQHCs provide primary care services on a sliding fee scale based on financial need regardless of a patient’s ability to pay. In 2023, the clinic served 53,824 patients across 120,818 medical visits, with 56% covered by Medicaid, 20% uninsured, 16% privately insured, and 7% enrolled in Medicare. The clinic operates four 340B-participating community pharmacies, offering reduced-cost medications, pharmacist-led home health services accredited by the AAAHC, and telehealth services. At the time of the study, no ASP was in place.

### 2.3. Questionnaire Development, Deployment, and Analysis

To develop the survey, we reviewed the CFIR Interview Guide Tool [8] and the work of Butler et al. (2021) [9] to refine the questions, modifying the language and selecting relevant items. Four pharmacists—three administrators and one clinical pharmacist—independently reviewed the final survey to ensure content relevance. Their evaluations were conducted independently of the research team, providing an unbiased assessment of the survey’s appropriateness and relevance. We created questionnaires (Supplementary Material S1) for both an electronic survey and focus groups, deploying the survey via the Qualtrics platform.

We purposively recruited those most likely to play a key role in ASP implementation. These include physicians, nurses, pharmacists, IT staff, and administrators. An anonymous survey was distributed to these participants, followed by three virtual focus groups conducted via Zoom. Given the diverse and demanding schedules of the participants, the focus groups were stratified into three groups: non-healthcare providers, healthcare providers, and a one-on-one interview with the Chief Medical Officer. A pharmacy resident conducted the sessions, reading from an interview question script specifically designed for this study. The audio recordings were analyzed by a qualitative researcher trained in thematic analysis, employing the CFIR framework to identify key constructs. NVivo Qualitative Data Analysis Software (Version 15) was used to ensure a systematic and rigorous analysis of the data.

## 3. Results

### 3.1. Quantitative Analysis

Out of over 40 clinical staff, 13 completed the survey (Table 1). Key potential patient-related barriers to ASP participation were wait times, medication adherence, and language issues, while potential staff-related barriers included patient pressure to prescribe antibiotics, a lack of ASP training, and a high workload.

Regarding ASP benefits, 53.8% strongly agreed and 23.1% somewhat agreed that ASP improves antibiotic prescribing practices. Similarly, 46.2% strongly agreed and 30.8% somewhat agreed on their commitment to ASP success. However, only 15.4% felt adequately trained in ASP, and 23.1% disagreed that leadership provided regular feedback. Finally, 69.2% were open to clinical process changes, while 23.1% remained neutral (Table 2).

### 3.2. Focus Group Interview

Our survey findings were thematically categorized (see Supplemental Table S1 for themes and illustrative quotes). The participants emphasized the need for personalized educational materials tailored to antibiotic stewardship programs (ASPs) in community health settings. Recommendations included videos featuring familiar staff, clinic uniforms, and logos to build trust and relatability. Pamphlets and digital campaigns were also suggested but required regular updates and significant resources to maintain engagement and effectiveness.

*Barriers to Implementation*: Focus groups highlighted key barriers to ASP implementation, such as the diverse physical layouts and geographic dispersion of clinics, which complicated communication and consistent program execution. Tracking patient outcomes, particularly for walk-in patients, was another significant challenge. Proposed solutions included robust communication systems, improved follow-up mechanisms, and new technology and training. However, these solutions were acknowledged to be resource-intensive and complex.

*Patient and Staff Challenges*: Staff identified limited patient understanding and technology barriers as obstacles to antibiotic education. They also faced significant pressure from patients who demanded antibiotics, necessitating clear protocols to manage these expectations. Participants advocated learning from successful ASPs in other facilities and enhancing the documentation of antibiotic use to monitor program success. The overarching goal was to reduce unnecessary antibiotic prescriptions, which required meticulous monitoring and substantial administrative efforts.

*Clinical Observations and Staff Feedback*: Clinicians noted that many patients requested antibiotics prematurely, often visiting the doctor after only one or two days of symptoms without trying over-the-counter remedies. Additionally, clinicians reported feeling pressured to prescribe antibiotics to satisfy patients, even when symptoms suggested a viral cause.

*Anticipated Outcomes*: Participants expected ASPs to result in a measurable decrease in antibiotic prescriptions. They noted that many walk-in clinic patients arrived with the expectation of receiving antibiotics and often expressed frustration when those expectations were unmet. Reducing unnecessary antibiotic use was identified as a key priority, but success depended on addressing these patient expectations through education and programmatic support.

## 4. Discussion

Our findings highlight the key contextual factors influencing the implementation of ASPs in a primary care setting serving rural or underserved communities. Based on our findings, the respondents consistently emphasized the potential positive impact of antibiotic stewardship in improving antibiotic use and expressed a deep commitment to their success when implemented. The perception that the implementation of ASPs can offer more benefits than drawbacks was particularly notable, with an emphasis on their advantages in outpatient care settings. These findings are significant because when clinicians perceive programs like antibiotic stewardship as valuable and effective, they are more likely to act as champions of these initiatives, promoting sustained behavior change and integration into organizational culture. As highlighted by Klaic et al. (2022) [10], a positive perception among clinical staff and clinicians enhances the implementability and scalability of ASPs, reinforcing the importance of aligning stewardship efforts with provider beliefs and organizational priorities to ensure long-term success.

However, while clinic leadership was perceived as generally supportive of antibiotic stewardship goals, some respondents were uncertain about leadership’s willingness to enforce compliance. Given that strong leadership commitment is a key driver of successful ASP implementation, these findings underscore the need for clear prioritization, accountability structures, and enforcement mechanisms to sustain meaningful change.

Furthermore, a notable gap was identified in staff education and training, with 38.5% reporting insufficient antibiotic stewardship training and 46.2% remaining neutral. This observation highlights the importance of educating healthcare professionals on antibiotic stewardship principles and adaptation. As part of education, we propose the development of treatment algorithms for common infectious diseases. All of these serve as foundational steps for implementing antibiotic stewardship.

Finally, we observed a strong sense of accountability for patient care and appropriate antibiotic use among clinical staff, with 84.6% expressing high responsibility for patient safety. Survey and focus group discussions revealed staff willingness to adopt ASPs, recognizing their potential to improve outcomes and combat antibiotic resistance. This openness underscores the need to foster awareness and buy-in for successful implementation.

Although these findings were drawn from a clinic serving rural or underserved populations, we believe similar challenges and facilitators could be observed in clinics serving urban communities. A 2020 cross-sectional multicenter survey of Vizient member hospitals with outpatient settings found that only 7% of ambulatory practices had implemented ASPs compared to 88% of inpatient member institutions—which are mostly in urban communities [11]. The observed low number of ambulatory services that implemented ASPs could be a consequence of limited resources, insufficient leadership support, lack of training on antibiotic stewardship, or competing priorities—all of which may hinder ASP implementation in outpatient settings regardless of geographic location [12,13]. Thus, our findings reinforce the need for targeted strategies to address barriers and promote ASP adoption across diverse healthcare settings.

### 4.1. Limitation

Our study has some limitations. The small sample size (low response rate), coupled with its focus on a single rural health center, limits the generalizability of the findings to other settings or populations. Additionally, since the survey was anonymous, it is unclear whether the four pharmacists who reviewed the questionnaires were also survey participants. However, they did not participate in the focus groups, ensuring that their involvement as reviewers did not influence the qualitative data obtained. A notable strength is that the focus group participants closely resembled the population of interest in rural healthcare settings, enhancing the relevance of the findings to similar environments.

Despite these strengths, the findings may not be directly transferable to other healthcare contexts, especially those in different countries with distinct professional, cultural, and healthcare systems. Such differences underscore the need for caution when applying these insights beyond similar rural healthcare settings. However, a key strength of this study is the use of the CFIR—an evidence-based framework that provided a rigorous structure for analyzing contextual factors. This methodological approach strengthens the study’s contributions to understanding antibiotic stewardship implementation in rural health centers, where resources are often limited but the need for effective interventions is critical.

### 4.2. Future Study

Based on the findings of this pilot study, future research should focus on conducting a multicenter study to further explore the generalizability of the results across diverse rural healthcare settings. Expanding the scope to include multiple rural health centers with varying professional, cultural, and healthcare system contexts would provide a broader understanding of the factors influencing antibiotic stewardship implementation.

## 5. Conclusions

This pilot study offers valuable insights into the specific contextual factors influencing ASP implementation within this rural healthcare setting. The findings highlight the strong commitment of healthcare providers and staff to improving antibiotic use and patient outcomes, with ASPs recognized as beneficial interventions in this context. However, challenges unique to this setting, including limited staff training, inconsistent leadership enforcement, and the constraints of rural healthcare environments, must be addressed to enhance the feasibility and success of ASPs. By leveraging the observed sense of accountability and willingness among healthcare staff to engage with stewardship initiatives, there is significant potential to advance ASP implementation within this pilot study’s focus area.

## Figures and Tables

**Table 1:** Demographics and Barriers to ASP Implementation.

Respondents’ Roles in Community Health Center	N = 13
Physician	3 (23%)
APRN (nurse practitioner)	2 (15%)
RN/LPN (staff nurse)	2 (15%)
Clinical staff	0
MA (medical assistant)	0
Pharmacist	5 (38%)
PA (physician assistant)	1 (8%)
**Potential patient-related barriers for ASP implementation **	
Longer wait times (time to discharge)	2 (17%)
Cost (cost of changing from one antibiotic prescription to another)	1 (8%)
Adherence to prescribed medication	3 (25%)
Staff shortages	1 (8%)
Transportation	1 (8%)
Others: Cost, adherence, transportation, language barrier, education on ASP	2 (17%)
**Potential clinic-related barriers for ASP implementation**	
High workload	4 (31%)
Time constraints	3 (23%)
Decision fatigue (diminishing quality of decisions after prolonged decision-making)	1 (8%)
Patient pressure (pressure from patients to prescribe antibiotics)	7 (54%)
Diagnostic barriers (inadequate diagnostic kits/equipment)	2 (15%)
Staff shortage	1 (8%)
Limited communication	3 (23%)
Limited access to patients’ information	2 (15%)
Lack of training and knowledge	8 (62%)
Lack of resources	2 (15%)
Other	1 (8%)

**Table 2:** Perceived Beliefs and Contextual Factors Influencing Antibiotic Stewardship Program Implementation.

Indicate the Level of Agreement with the Following Statements Regarding Antibiotic Stewardship Programs (ASP) [N = 13]	Strongly Disagree	Somewhat Disagree	Neither Agree or Disagree	Somewhat Agree	Strongly Agree
Research shows that ASPs effectively improve antibiotic prescribing.	0 (0%)	(7.7%)	2 (15.4%)	3 (23.1%)	**7 (53.8%)**
We have received enough education and training on the ASP.	3(23.1%)	2 (15.4%)	**6 (46.2%)**	2 (15.4%)	0 (0%)
I am committed to the success of the ASP.	0 (0%)	0 (0%)	3 (23.1%)	4 (30.8%)	**6 (46.2%)**
Clinical leadership regularly provides staff with feedback/data on the effects of clinical decisions.	0 (0%)	3 (23.1%)	**6 (46.2%)**	3 (23.1%)	1 (7.7%)
Based on clinical experience, ASPs prove to be beneficial in improving antibiotic prescribing.	0 (0%)	1 (7.7%)	2 (15.4%)	**6 (46.2%)**	4 (30.8%)
ASPs add value to our organization.	0 (0%)	1 (7.7%)	2 (15.4%)	**5 (38.5%)**	**5 (38.5%)**
ASPs will offer more benefits than drawbacks for outpatients.	0 (0%)	1 (7.7%)	1 (7.7%)	5 (38.5%)	**6 (46.2%)**
Clinic leadership will set a high priority on the success of the ASP to improve antibiotic prescribing.	0 (0%)	1 (7.7%)	4 (30.8%)	**6 (46.2%)**	2 (15.4%)
Clinic staff feel a personal responsibility for enhancing patient care and outcomes.	0 (0%)	0 (0%)	2 (15.4%)	**8 (61.5%)**	3 (23.1%)
Clinic staff are open to changes in clinical processes.	1 (7.7%)	0 (0%)	3 (23.1%)	**7 (53.8%)**	2 (15.4%)
I view ASPs as one of the important interventions the clinic can adopt.	1 (7.7%)	0 (0%)	1 (7.7%)	4 (30.8%)	**7 (53.8%)**
Clinical leadership is willing to give the antibiotic steward committee the authority to enforce the ASP policies.	1 (7.7%)	1 (7.7%)	4 (30.8%)	**5 (38.5%)**	2 (15.4%)

## Data Availability

The original contributions presented in this study are included in the article/Supplementary Materials. Further inquiries can be directed to the corresponding author.

## Supplementary Materials

The following supporting information can be downloaded at: https://www.mdpi.com/article/10.3390/antibiotics14030263/s1, Supplementary Materials S1. Survey Questionnaire. Supplementary Materials S2. Table S1. Focus Group Interview.
