# Supplementary material for: A Pilot Study on Understanding the Contextual Factors Impacting the Implementation of an Antibiotic Stewardship Program in a Single Health Center Serving Rural and Underserved Communities in the United States—A Mixed-Methods Approach
Source: Antibiotics (Basel). 2025 Mar 5;14(3):263. doi: 10.3390/antibiotics14030263 (PMC11939229; doi:10.3390/antibiotics14030263)
Supplement: Supplementary file 1 [file antibiotics-14-00263-s001.zip › antibiotics-3397052-Supplementary S1.pdf]

A Pilot Study on Understanding the Contextual Factors Impacting the Implementation of Antibiotic Stewardship Program in a Single Health Center Serving Rural and Underserved Communities in the United States – A Mixed Method Approach

Survey Questionnaire

Introduction to be read by the respondents prior to completing the survey:

"The implementation of an antibiotic stewardship program in the outpatient clinic is now required by the Joint Commission. Antibiotic stewardship involves measuring and enhancing how clinicians prescribe antibiotics and how patients use them. Improving antibiotic prescribing and use is critical for effectively treating infections, safeguarding patients from the harms of unnecessary antibiotic use, and combating antibiotic resistance. We would like to assess your opinion on the implementation of antibiotic stewardship (ASP) at your clinic."

Survey Questions:

What is your role at the hospital, [Physician, ARNP, RN, Clinical Staff, IT staff, Other ----------]

Please indicate the level of agreement with the following statements regarding Antibiotic stewardship program (ASP) below

Strongly Disagree
Disagree
Neutral
Agree
Strongly Agree

1. Research shows that ASPs effectively improves antibiotic prescribing.
2. Based on clinical experience, ASPs prove to be beneficial in improving antibiotic prescribing
3. ASPs add value to our organization.
4. ASPs will offer more benefits than drawbacks for our patients.
5. Clinic leadership will set a high priority on the success of the ASP to improving antibiotic prescribing.

6. Clinic staff feel a personal responsibility for enhancing patient care and outcomes.

7. Clinic staff are open to changes in clinical processes.

8. I view ASPs as one of the important interventions the clinic can adopt.

9. Clinical leadership is willing to give the antibiotic steward committee the authority to enforce the ASP policies.

10. We have received enough education and training on the ASP.

11. I am committed to the success of the ASP.

12. Clinical leadership regularly provides staff with feedback/data on the effects of clinical decisions.

<u>Focus Group Interviews/ Written</u>

1. What barriers do the patients served by your clinic face to participating in the ASP?

2. What barriers do the staff of your clinic face in participating in the ASP?

3. What kind of local, state, or national performance measures, policies, regulations, or guidelines influenced the decision to implement the ASP?

4. How will the infrastructure of your clinic (social architecture, age, maturity, size, or physical layout) affect the implementation of the ASP?

5. How will the infrastructure facilitate or hinder implementation of the ASP?

6. What else is on your mind regarding what will facilitate or hinder the implementation of ASP in the clinic?
