# Supplementary material for: A Pilot Study on Understanding the Contextual Factors Impacting the Implementation of an Antibiotic Stewardship Program in a Single Health Center Serving Rural and Underserved Communities in the United States—A Mixed-Methods Approach
Source: Antibiotics (Basel). 2025 Mar 5;14(3):263. doi: 10.3390/antibiotics14030263 (PMC11939229; doi:10.3390/antibiotics14030263)
Supplement: Supplementary file 1 [file antibiotics-14-00263-s001.zip › antibiotics-3397052-Supplementary S2.pdf]

**Focus Group Interview**	
**Themes**	**Selected Specific Quotes**
Patient Education for Antibiotics	"There's a media screen in our waiting area that kind of plays the same looped video… We can make a video, but don't use one off the Internet. We want to make one that has our uniforms, our logo and our familiar faces with the patient…a video talking about the importance of the overuse of antibiotics and the importance of completing antibiotics from people in our clinic."
Barriers to Implementation of ASP in Community Health	"I guess if we want to talk about the physical layout, I know sometimes in this department it is hard to support clinicians and staff, just because everybody's at different locations with different times or covering different sides. So it's hard to keep up with the changes."<br>"… and it is hard to communicate between other staff members at different sites. I do have extensions, but I'm rarely, if ever, at my desk for more than five minutes at a time. So it is hard to communicate with not only the patients but staff as well…"<br><br>"I'm not 100% sure what the turnaround is for patients at walk in [walk-in clinic], but I would think that following up would be a little bit harder with the once the antibiotic regimen is completed…<br><br>"...But just previous experience, that other facility was kind of hard to follow patients."<br><br>"…we may see them and never see them again. We try to encourage them to, of course, follow up with primary, but a lot of times they can't get in for months. And then they might go to another clinic to follow up. So it is hard to ensure people are getting better and don't need another antibiotic and things like that."
Patient Barriers to Participation in ASP	"...So how will this stewardship program be presented to the patients? Because that could be an issue as far as communication and technology."
Patient Barriers with Antibiotics	"…A lot of patients come in and want antibiotics too soon. They haven't tried any over the counter regimens first and they've only had symptoms for 1 or 2 days. So I think there's a knowledge deficit there."<br>"…I think sometimes maybe the clinicians feel pressured to give something to the patient to make them feel better…"
Staff Barriers to Participation in ASP	"A lot of times patients come in here and they get quite upset when we don't fulfill their requests for antibiotics that aren't warranted…"<br>"Maybe some type of protocol in place for a walk in? Let's say that when the patients are being triaged, before they even get back to the provider that says, you know, have you asked the patient X, Y, I mean, have they taken anything for the symptoms? How long have the symptoms been and maybe have a cut off. Well, they've been sick for a week."<br>"Oh, yeah. We'll see them. But if they've been sick for two days, maybe have that protocol in place."
Suggestions for Implementing ASP in the Clinics	"I think I will kind of want to know or see examples of the stewardship program at other facilities, how they implement it, how it's going. Just experiences about other facilities will definitely be helpful."<br>"I feel like we as providers and staff need to document when we used, the templates that were given to us because we have used it twice today, both for chronic sinus management. So I wonder if there's a way that we can document which patients we're using it for and why. Or maybe we can have like a spreadsheet to chart that in a way. I'm not sure if that's helpful at all."<br>If the providers had a way to just point and click to something that's already set up in the computer, when that applies not applicable."
Outcomes for ASP	"…definitely a decrease in antibiotic use. Like I said, a lot of times patients come to walk in and their expectation is that they will always get an antibiotic and they're quite upset when that does not happen. So a decrease in prescriptions is probably what I'm in favor of."
